# Risk Factors Related to the Development of Nonalcoholic Fatty Liver: A Systematic Review

**DOI:** 10.1155/cjgh/9964486

**Published:** 2025-04-15

**Authors:** Omaira Valencia, Carolina López, Esteban Vanegas-Duarte, Carolina Fillizola, Diana Fernanda Bejarano Ramírez, Nicolás Andrés Cortés Mejía, Alonso Vera Torres

**Affiliations:** ^1^Population Health, Fundación Santa Fe de Bogota, Bogota, Colombia; ^2^Transplant and Hepatobiliary Surgery Department, Fundación Santa Fe de Bogotá, Bogotá, Colombia; ^3^Epidemiology and Biostatistics Group, Graduate School of Epidemiology and Biostatistics, CES University, Medellín, Colombia; ^4^Division of Anesthesiology, Critical Care Medicine and Pain Medicine, The University of Texas MD Anderson Cancer Center, Houston, Texas, USA

**Keywords:** NAFLD, nonalcoholic fatty liver disease, risk factors

## Abstract

**Background:** Nonalcoholic fatty liver disease (NAFLD) has a major impact on public health owing to its high morbidity and mortality due to its close relationship with several conditions, including metabolic syndrome, cirrhosis, and cancer. Therefore, this review aimed to systematically compile and summarize the scientific literature on early risk factors for NAFLD development.

**Methods:** A systematic review of population-based cohort studies was conducted. Studies reporting the risk factors associated with nonalcoholic steatohepatitis (NASH) and NAFLD were screened.

**Results:** The search yielded 987 unique records, of which 196 articles were selected after title and abstract screening. A total of 39 articles were read in full text after quality analysis using Downs and Black criteria; 10 of the studies were excluded due to heterogeneity or inconclusive results. Finally, 30 publications were included in this systematic review. The review revealed that clinical conditions such as obesity, weight change, psoriasis, polycystic ovary syndrome, diabetes, thyroid disorders, and elevated serum uric acid levels increase the risk of developing nonalcoholic fatty liver. In addition, lifestyle factors such as sedentary behavior, active or passive smoking, poor sleep quality, and consumption of carbonated beverages are associated with this condition.

**Conclusions:** Evidence was found on the association between different clinical and lifestyle risk factors and NAFLD. This supports the need for preventive, diagnostic, and therapeutic strategies to improve the metabolic, hepatic, and oncological outcomes related to this condition.

## 1. Background

Hepatic steatosis or fatty liver is a significant public health issue owing to its association with metabolic diseases such as diabetes mellitus (DM), obesity, and metabolic syndrome (METS). They are recognized as major causes of morbidity and mortality. Hepatic steatosis may occur secondary to nonalcoholic fatty liver disease (NAFLD), alcoholism, chemotherapy, toxicity, and infectious agents. Although NAFLD is asymptomatic in its initial stages, it leads to an increased incidence of hepatic and nonhepatic comorbidities, including cirrhosis, hepatocellular carcinoma, cardiovascular disease, and other metabolic disturbances [1]. This is particularly true in developed countries [2]. Notwithstanding, the burden of metabolic diseases (type 2 DM and fatty liver) has increased worldwide at all levels of development [3].

NAFLD has many spectra, ranging from simple fat accumulation with no symptoms (NAFLD) to symptomatic nonalcoholic steatohepatitis (NASH). Simple steatosis has a silent and benign course in the early stages; however, if it progresses to NASH, it can lead to liver cirrhosis and hepatocellular carcinoma [4]. Genetically susceptible individuals under adverse environmental conditions are prone to developing NAFLD. Intrahepatic lipid accumulation induces the production of reactive oxygen species, leading to local and systemic inflammation that progressively evolves into NASH. If these processes persist, interstitial collagen deposition and fibrosis can occur, leading to cirrhosis. A persistent inflammatory microenvironment favors cancer development [5]. In these advanced scenarios, the only viable treatment is liver transplantation (LT) [6].

It is estimated that more than a quarter of the population has NAFLD. In Latin America, the prevalence of NAFLD ranges from 17% to 33.5% [7], and NASH accounts for 75% of all chronic liver diseases. The global prevalence of NAFLD is estimated to be 25% in the general population. Most epidemiological studies on NAFLD have been conducted in the United States, where the prevalence is 24.13%. African Americans had the lowest prevalence, followed by European Americans and Hispanic Americans. Despite the ability to diagnose NAFLD through noninvasive methods, the exact prevalence of NASH in the general population remains unknown because, to date, only liver biopsy can precisely confirm the diagnosis, and indirect data suggest that the prevalence of NASH in the general population ranges from 3% to 5%, using estimates from American population data [8].

In the United States, healthcare related to liver disease care is the most expensive, with an estimated total cost of 13.1 billion dollars (80.6% indirect costs and 19.3% direct costs) [9]. According to data from the European Liver Transplant Registry (ELTR) and the United Network for Organ Sharing (UNOS) databases, NAFLD has been the fastest-growing transplant indication in the last 20 years. It is currently the second leading cause of transplant-requiring liver disease, present in 1321 (21.5%) adult patients who underwent LT in 2018. A similar trend was observed in European countries, where NAFLD-related cirrhosis accounted for 1.2% of LT in 2002 and 8.4% in 2016 [10].

To control the progression from NAFLD to NASH, it is essential to understand the underlying mechanisms. Therefore, this review aimed to systematically compile and summarize the scientific literature on early risk factors for the development of NAFLD.

## 2. Methodology

This systematic review was conducted and reported following the Preferred Reporting Items for Systematic Reviews (PROSPERO) (CRD42022357262) and Meta-Analyses (PRISMA) statement. Analytical observational data that included social, demographic, and clinical risk factors associated with NAFLD development in the adult and pediatric populations were analyzed. The following databases were consulted to identify indexed publications: the Cochrane Central Register of Controlled Trials (CENTRAL), the Cochrane Library, MEDLINE through the PUBMED search engine, and Latin American and Caribbean Health Sciences Literature (LILACS). In addition, snowball searches were conducted based on references from published publications or those provided by clinical experts. The protocol for this systematic review is registered in the International Prospective Register of Systematic Reviews (PROSPERO).

### 2.1. Search Strategy

Key terms in natural languages from the research questions in the PICOT format were used to construct the search strategy. The search syntax was composed of controlled vocabulary, such as Medical Subject Headings (MeSH) terms for Medline and Cochrane engines and DeCS terms (Health Science Descriptors) for the LILACS engine. Free terms were also used, considering synonyms, abbreviations, acronyms, and spelling variations, and were complemented with field identifiers, truncators, and Boolean operators. Details of the search strategies used and their results are presented in Appendix A. The list of bibliographic references obtained through the described methods was imported into Mendeley, where references were combined and duplicate publications were removed.

### 2.2. Study Selection

Studies were chosen following the PRISMA flowchart (Matthew J. Page, 2021), with the assistance of the systematic review software Rayyan (https://rayyan.qcri.org) [11]. The refinement process began with duplicate detection using Rayyan software. After eliminating duplicated results, two independent authors (CL and NL) reviewed the titles and abstracts to identify studies eligible for full-text review. In the case of discrepancies, the evaluation criteria were revisited. Subsequently, a third evaluator reviewed all articles to reduce any bias in the initial review by the initial evaluators. After this process, full-text reading was conducted independently by two evaluators to determine whether each study should be included or excluded from evidence synthesis. Discrepancies were resolved through consensus among the evaluators, and if disagreements persisted, a third evaluator was consulted.

Studies with five or fewer patients with critical results were excluded as they were considered to have insufficient reliability. Case reports, case series, and systematic literature reviews were excluded. Articles lacking complete information about the type of patient, exposure factors to be evaluated, inclusion and exclusion criteria, or outcomes were also excluded. Downs and Black's criteria [12] were used to assess the quality of the studies; they evaluated report quality (10 items), external validity (3 items), internal validity (bias: 8 items and confusion: 6 items), and statistical power (1 item). In addition, this instrument assessed study biases. Articles with a score of ≥ 80% were defined as high quality and were included. Evaluation was independently conducted by two methodologists (CL and NL); in case of discrepancies in the initial assessment of any of these criteria, resolution occurred through consensus among evaluators.

### 2.3. Data Extraction

For each included study, information was extracted in a standardized format. The qualitative synthesis of articles included information about the authors, study design, location, period, restrictions on patient selection, and study results. Patient characteristics, including sex, age, body mass index (BMI), clinical and/or pathological history, and the method of detecting hepatic steatosis, were collected. Extraction was independently performed by two reviewers (CL and NL). This information serves as the basis for constructing a body of evidence.

### 2.4. Data Analysis

For each study, data were extracted in a standardized format: author, country, publication date, population, risk factors for NAFLD, and age. The extraction was performed independently by two reviewers. In each study, according to the statistical analysis, reported risk measures (odds ratio [OR], relative risk [RR], or hazard ratio [HR]), and confidence interval (CI) for each risk factor were included. The extraction was performed independently by two reviewers. The results were presented narratively, indicating the main findings for each included article based on the identified risk factors.

## 3. Results

**Figure 1:**
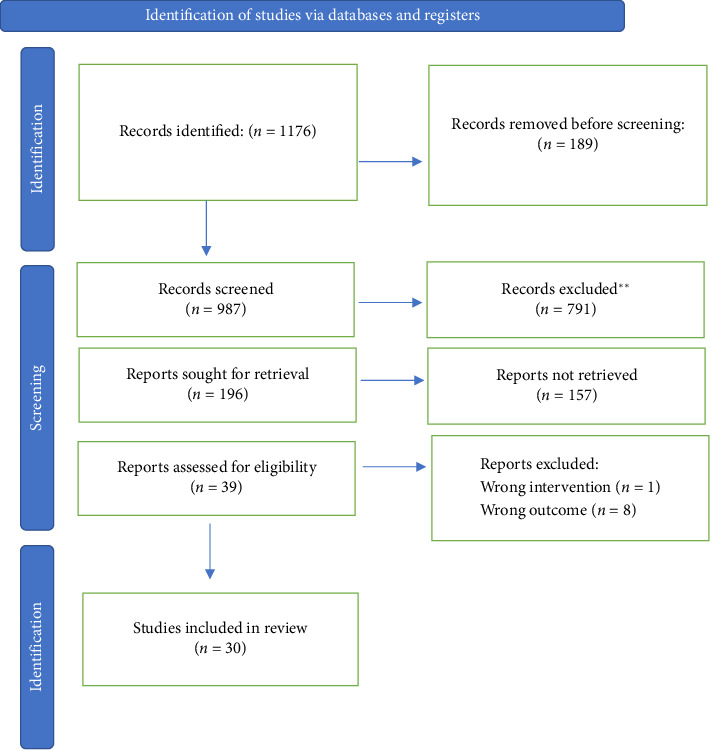
PRISMA flowchart.

Figure 1 displays a PRISMA flowchart depicting the article selection process. The search yielded 987 records. A total of 196 manuscripts were selected after screening the titles and abstracts. Downs and Black quality analysis yielded 39 articles for full-text reading. Of these, 9 studies were excluded due to heterogeneity or inconclusive results. Ultimately, 30 publications were included in this systematic review (Figure 1).

Studies from Korea, Japan, the United States, Turkey, Brazil, the Netherlands, Germany, Sweden, the United Kingdom, Spain, and China were included, comprising data on over 21 million individuals. Among the eligible studies, twelve had a prospective cohort design, two had a retrospective cohort design, nine were cross-sectional, and six were case-control studies. All study samples were community-based general populations and some were defined through data linkage.  Table 1 presents the risk factors identified in the review process. In 21 articles, the study population consisted of middle-aged adults (18–60 years); six included young adults and adolescents, and two involved middle-aged adults and school-age individuals. The most commonly used method for detecting hepatic steatosis was ultrasound, which was used in 22 of the included studies.

### 3.1. Clinical Conditions

Four studies assessed weight change, overweight, or obesity as prognostic factors in adults and two studies in adolescents. VanWagner et al. [13] evaluated BMI change patterns over 25 years in a prospective cohort of 4423 patients aged 18–30 years. They found that increasing BMI changes had progressively higher odds of NAFLD compared with the stable group: OR: 3.35 (95% CI: 2.07–5.42), 7.80 (4.60–13.23), and 12.68 (6.68–24.09) for moderate, high, and extreme increases, respectively. Kim et al. [14] prospectively collected data from 110,054 women, revealing that women with ≥ 20 kg weight gain in adulthood had a multivariable adjusted HR (aHR) of 6.96 (5.27–9.18) for NAFLD development, which remained significant after BMI adjustment. Cho et al. [16] conducted a longitudinal cohort study in 1907 participants and found that the risk of incident NAFLD was significantly higher in subjects with ≥ 10% weight gain (HR: 2.43; 1.65–3.58). Regarding the adolescent population, Boyraz et al. [17] conducted a prospective cohort study in 451 obese pubertal children (8–18 years old). Among them, 217 (48.1%) were diagnosed with NAFLD, and 96 (21.3%) were diagnosed with METS. The prevalence of METS is higher in patients with NAFLD. In addition, the number of patients with METS increased in parallel with the severity of hepatic steatosis [17].

Gestational diabetes mellitus (GD) has also been identified as an independent risk factor for NAFLD. Ajmera et al. evaluated 1115 women without DM before pregnancy from the Coronary Artery Risk Development in Young Adults (CARDIA) multicenter cohort study; these patients underwent hepatic steatosis quantification using computed tomography (CT) 25 years after cohort entry. The crude risk of NAFLD at the 25-year visit was significantly higher in women with gestational diabetes than in those without gestational diabetes (OR: 2.56 and 95% CI: 1.44–4.55). In these studies, remaining overweight was associated with a lower chance of resolving steatosis [22].

Lavrentaki et al. conducted a retrospective matched-cohort study using the Health Improvement Network (THIN), a large database of 9640 women with GD and 31,296 healthy controls. The unadjusted incidence rate for NAFLD development in women with and without GD was 3.28 (95% CI: 2.14–5.02), which remained significant after adjustment for a wide range of potential confounding factors. Finally, the Swedish ESPRESSO cohort study found that maternal BMI was associated with NAFLD in offspring (OR 3.26; 95% CI: 1.72–6.19) [23].

The relationship between serum uric acid level and NAFLD was also investigated. A prospective cohort study, including 2832 subjects without NAFLD, analyzed four groups based on initial serum uric acid levels. Cox regression analysis revealed that the RR for NAFLD was 1.431 (95% CI: 1.123–1.823), 1.610 (95% CI: 1.262–2.054), and 1.666 (95% CI: 1.287–2.157) in the second, third, and fourth quartiles of serum uric acid, respectively, adjusted for other confounding factors. Kaplan–Meier analysis revealed that individuals with higher uric acid levels had a higher risk of NAFLD than those with lower uric acid levels [33]. Similarly, a cross-sectional study with subsequent longitudinal follow-up analyzed uric acid levels in 60,455 subjects according to sex-specific quartiles. The ORs for NAFLD in the cross-sectional population were 1.211 (95% CI: 1.109–1.322), 1.519 (95% CI: 1.395–1.654), and 1.903 (95% CI: 1.748–2.072) for the second, third, and fourth quartiles, respectively. In the longitudinal population, compared with the reference group, those in q2, q3, and q4 had HR of 1.127 (95% CI: 0.956–1.330), 1.380 (95% CI: 1.157–1.644), and 1.589 (95% CI: 1.310–1.927) for NAFLD, respectively [34].

In addition, the risk of developing NAFLD in individuals with thyroid disorders has been investigated. A German case-control study compared 57,483 patients with NAFLD with 57,483 patients without liver disease. Hypothyroidism (OR: 1.17 and 95% CI: 1.10–1.24) and autoimmune thyroiditis (OR: 1.53 and 95% CI: 1.35–1.73) were associated with a higher risk of NAFLD [25]. Chen et al. conducted a cohort study with 3144 euthyroid subjects, of which 2089 were followed up after 2.2 years. The age-adjusted mean levels of FT3 and the FT3/free thyroxine (FT4) ratio were higher in subjects with NAFLD than in those without NAFLD [43].

Xu et al. conducted a cross-sectional study of 439 patients with psoriasis. The prevalence of NAFLD in this cohort was 55.8%. NAFLD was frequently identified in early onset patients (74.2%), and this diagnosis is particularly common in patients under 40 years of age (85.3%) [44]. In addition, a case-control study by Ogdie et al. found that the risk of cirrhosis was higher in patients with psoriasis and psoriatic arthritis. Furthermore, the prevalence of liver disease and cirrhosis gradually increases as the body surface area affected by psoriasis increases [28]. Busca Arenzana et al. conducted a retrospective observational study on HIV-positive patients diagnosed with psoriasis. In a cohort of 80 patients with psoriasis, the prevalence of steatosis was 72.5% (95% CI: 65–85). Severe psoriasis was an independent risk factor for steatosis (OR: 12 and 95% CI: 1.2–120) [29].

Polycystic ovary syndrome (PCOS) is associated with NAFLD onset. A case-control study identified that patients with PCOS had a significantly higher rate of NAFLD (OR: 4.30 and 95% CI: 4.11–4.50) [30]. In another cross-sectional study that analyzed 29 women with PCOS, patients with hyperandrogenic PCOS had significantly higher hepatic fat than patients with normoandrogenic syndrome (OR: 3.7%; 95% CI: 0.6–13.1) and controls (OR: 2.1%; 95% CI: 0.3–6.6) [31].

### 3.2. Lifestyle

One study reported an association between poor sleep quality and the onset of NAFLD. This cross-sectional study assessed sleep quality of 69,463 middle-aged workers and their spouses. After controlling for confounding factors, the adjusted OR for NAFLD, comparing sleep duration less than 5 h (compared with the reference of more than 7 h), was 1.28 (1.13–1.44) in men and 1.71 (1.38–2.13) in women. After adjusting for BMI, this association remained significant in women (OR: 1.59 and 95% CI: 1.23–2.05) [41].

Regarding dental hygiene habits, a retrospective longitudinal study in 25,804 individuals showed that brushing the teeth 1–2 times a day (OR: 0.85 and 95% CI: 0.77–0.95) and 3 times a day (OR: 0.74 and 95% CI: 0.67–0.82) is a protective factor for the development of NAFLD [36]. A prospective cohort study investigated the association between sugary carbonated beverage consumption and NAFLD in 14,845 participants. The multivariable HR for NAFLD incidence was 1.00 for < 1 serving/week of carbonated beverage, 1.18 (95% CI: 1.03–1.34) for 1 serving/week of carbonated beverage, 1.23 (95% CI: 1.08–1.40) for 2–3 servings/week of carbonated beverage, and 1.47 (95% CI: 1.25–1.73) for ≥ 4 servings/week of carbonated beverages [40].

A study that included 79,048 Korean adults without NAFLD evaluated the relationship between fatty liver and weekly working hours in the ranges of 35–40, 41–52, 53–60, and > 60 h. During a median follow-up of 6.6 years, 15,095 participants developed new-onset NAFLD. After adjusting for confounding factors, the RR for NAFLD development in 41–52, 53–60, and > 60 working hours compared with 35–40 working hours were 1.07 (95% CI: 1.02–1.13), 1.06 (95% CI: 1.00–1.13), and 1.13 (95% CI: 1.05–1.23), respectively [45]. Furthermore, a cross-sectional study based on the Korean National Health and Nutrition Examination Survey VII included 5661 working adults without liver disease, classified into three groups according to working hours: 36–42, 43–52, and 53–83 h per week. The prevalence of NAFLD (Hepatic Steatosis Index [HIS] ≥ 36) increased with longer working hours: 23.0%, 25.6%, and 30.6% in the 36–42, 43–52, and 53–83 h/week groups, respectively (*p* < 0.001). Subjects working 53–83 h per week were more likely to have NAFLD than those working 36–42 h per week (OR: 1.23, 95% CI: 1.02–1.50, and *p*=0.033) after adjusting for age, sex, BMI, smoking, alcohol consumption, exercise, diabetes mellitus, hypertension, serum triglycerides, and total cholesterol [38].

Tobacco enhances NAFLD by impairing fatty acid synthesis, IR, inflammation, and glucose metabolism [42]. A study found that smoking was related to NAFLD with an HR of 1.988 (95% CI: 1.057, 3.595) in 3860 participants; the HR increased as the number of cigarettes increased. In that study, the prevalence of NAFLD was 10% in those without tobacco exposure, 25% in those who smoked 1–14 cigarettes daily, and up to 30% in those who smoked more than 25 days [42].

### 3.3. Risk of Bias

The risk of bias in the sample was assessed using the Downs and Black Checklist [12]. Once this instrument was applied and the individual ratings of the 39 studies for each item were obtained, the percentage of studies that met each item was calculated for each subscale. In this way, the results obtained in the different subscales show that the external validity and internal validity of the sample were adequate for 29 articles, which were finally analyzed for the systematic review, and 10 articles were excluded due to heterogeneity in the findings.

## 4. Discussion

In this systematic review of 29 studies including over 21 million people, we addressed early risk factors for NAFLD development. Several clinical, metabolic, hormonal, inflammatory, and lifestyle features are associated with the onset of NAFLD. NAFLD and METS share pathophysiological pathways, which explain why they have several common risk factors, both of which are triggered by IR and systemic inflammation [1]. In up to 73% of the cases, both entities coexist, although some authors proposed NAFLD as an early marker of METS [46, 47].

Central obesity and overweight are classically associated with NAFLD [3]. However, there are different assessments for estimating the risk of NAFLD beyond BMI [13, 14]. The 2017-2018 National Health and Nutrition Examination Survey found that, after 10 years, a weight increase larger than 9.07 kg increases the odds of NAFLD (OR: 2.63 and 95% CI: 1.72–4.02) and that, for every kilogram weight increase, this risk increases by 3% [47]. This review found that DM facilitated NAFLD. Similarly, a meta-analysis including 3901 patients with type I DM found that 19.1% of them had NAFLD [48]. IR is crucial for NAFLD: when type II diabetics are compared with healthy controls and type I diabetics, they are found to have NAFLD more often (62.8% vs. 13.4% vs. 4.7%, respectively). Interestingly, in type I diabetes patients, NAFLD has been identified in those with uncompensated disease and overweight/obesity [49].

An increased BMI at conception, excessive weight gain, or GD increases the odds of NAFLD [22, 50, 51]; however, causality is not always determined, as approximately 10% of women of childbearing age have NAFLD prior to pregnancy [51, 52]. Furthermore, children whose mothers develop NAFLD during pregnancy are also at risk of developing this condition, especially if their mothers have abnormal periconceptional BMI, METS, abnormal weight gain after pregnancy, early pregnancy, tobacco exposure during pregnancy, or if the person had low weight at birth [50, 53, 54].

People from developed countries have increased exposure to screens [55]; however, this also occurs in developing countries [36]. In conjunction with increased working time, screen exposure is associated with NAFLD because it is related to poor feeding habits; omitting breakfast; eating out of time; instant food, alcohol, and tobacco consumption; overfeeding; chronic stress; and sleep deprivation [56]. Similarly, poor sleep quality may cause NAFLD as it enhances IR and inflammation [57]. In a survey of 143,306 healthy people, poor sleep quality was observed in 20.1% of the cases; in this group, 45.8% slept less than 5 h daily. After a 4-year follow-up, the HR for NAFLD was 1.19 if a person sleeps for less than 5 h [57]. Similarly, a Japanese study of 2172 healthy individuals found an NAFLD prevalence of 29.6%; higher rates were found in those who slept less than 6 h daily (OR: 1.44 (CI 95% = 1.06–1.96)) [58]. Interestingly, another study suggested that this effect could be mitigated if people catch up on weekends [59].

Tobacco enhances NAFLD by impairing fatty acid synthesis, IR, inflammation, and glucose metabolism [42]. In this review, a study found that smoking was related to NAFLD with an HR of 1.988 (CI 95%: 1.057 ± 3.595) in 3860 participants and that the HR increased as the number of cigarettes increased. Furthermore, a meta-analysis of 20 observational studies involving 20,149 patients found that smoking had an OR of 1.11 (CI 95%: 1.028–1.199), while passive smoking had an OR of 1.380 (CI 95%: 1.199–1.588). Unfortunately, smoking cessation did not prevent the onset of NAFLD (OR = 1.316 and CI 95%: 1.158–1.496) [60].

With regard to chronic inflammatory conditions, we found that psoriatic patients had an increased prevalence of NAFLD. When compared with the general population, the prevalence of NAFLD in this population is 17%–66% vs. 8%–35% [61]. It also causes more severe steatosis, fibrosis, and progression to NASH [61]. Moreover, HIV coinfection magnifies this effect [62], mainly because antiretroviral therapy and the virus itself impair lipid metabolism [63, 64].

Lastly, this review revealed that hormonal disturbances increase the risk of NAFLD. A metanalysis of 44,140 patients with thyroid disturbances found that clinical hypothyroidism was related to NAFLD, whereas subclinical hypothyroidism was not [65]; other meta-analyses that included 61,548 patients affirms that both entities increased NAFLD risk [2]. Similarly, NAFLD is 2–3.5 more common and severe in women than in men. This is explained in part by the fact that sex hormones play key roles in lipid and protein metabolism. In a study of 159 men with NAFLD, 26% had decreased testosterone levels. A decrease in this hormone level was also more frequent in patients with NASH (88% vs. 67%) and advanced fibrosis (27% vs. 14%) [67]. Interestingly, androgen excess, rather than deficiency, causes adverse effects in women. This is supported by a meta-analysis in which androgen levels of 13,721 men and 5840 women were compared, finding that androgens were markedly decreased in males with NAFLD, whereas they were increased in women with steatosis they were increased [66]. This also explains why women presenting with PCOS have NAFLD more often than the healthy population (37%–70% vs. 14%–34%) (OR = 3.93 and 95% CI: 2.17–7.11) [67]. The prevalence of NAFLD in women with PCOS is estimated to be 27%–62% [68]. Obesity is not a necessary factor for NAFLD in PCOS but may exacerbate the effects of PCOS on the liver [69].

This study had several limitations. First, it should be noted that some studies, but not all, focused on data from the general population, which allows the results to be applicable to unselected populations in some cases. Second, the studies were heterogeneous in design, definitions, and type of measurement. Each study set different thresholds for NAFLD diagnosis according to diagnostic technique (biopsy, ultrasound, CT, and MRI). Third, in most cases, coexistence rather than causality was described. Fourth, other potential risk factors for NAFLD were not included in this review because the study design did not match the eligibility criteria.

## 5. Conclusion

Despite this, this review summarizes substantial evidence on the association between different clinical and lifestyle risk factors and NAFLD, supporting the need for a more structured approach for the prevention and detection of nonalcoholic liver disease. Understanding these early risk factors is crucial for proposing prompt preventive and therapeutic interventions in exposed patients so that further major morbidities such as NASH and cirrhosis could be prevented. Given the increasing prevalence of obesity, unhealthy lifestyles, and METS, particularly during early ages, it is essential to develop screening strategies and follow-up starting in childhood.

This could be accomplished through public health policies that promote healthy lifestyles and follow-up, particularly in vulnerable populations who lack access to proper healthcare programs. Longitudinal studies and population-based follow-up focusing on how to effectively intervene in NAFLD risk factors should be proposed so that their results can guide the development of national policies aimed at preventing chronic conditions.

## Figures and Tables

**Table 1:** Characteristics of the studies included in the review.

Main risk factor	Added risk factor	Age group	Type of study	*n*	OR/HR/RR	Confidence interval 95%	Diagnostic method used	References
Weight variability	Increase in BMI > 10%	Young adult (18–30 years)	Cohort studies	4423	OR: 3.35	2.07–5.42	CT scan	[13]
								Korea
	Weight gain > 6 kg, regardless of BMI classification.	Adults: middle age	Cohort studies	110,054	HR: 1.59	1.17–2.16	Ultrasound	[14]
	Increase in visceral adiposity, measured by waist–hip ratio and BMI	Adults: middle age	Cohort studies		HR: 1.18	1.13–1.24		EE UU
	Weight gain > 3 kg in the short term (2 years).	Adults: middle age	Cohort studies	27,064	M. HR: 2.00	M. 1.90–2.12	Ultrasound	[15]
					F. HR: 1.92	F. 1.83–2.01		Japan
	Weight gain > 7%, regardless of the BMI range long term: 5 years.	Adults: middle age	Cohort studies	1907	HR: 2.43	1.65–3.58	Ultrasound	[16]
					*p* < 0.001			Korea
	Morbid obesity and diabetes	Adolescents	Cases and controls	590	OR: 6.77	2.72–16.89	Ultrasound	[17]
	Morbid obesity and hypertension				OR: 2.28	1.16–4.45		Turkey
					*p* < 0.02			

Obesity in adolescence	Morbid obesity and diabetes	Adolescents	Cohort studies	242	OR: 6.77	2.72–16.89	Ultrasound	[18]
								EE UU
	Diabetes mellitus type 1 and dyslipidemia		Cross-section	103	OR: 1.01	1.00–1.02	Ultrasound	[19]
					*p* 0.012			Brazil

Consumption of 2 drinks/day with sugar independent of BMI	No other associated factors	Childhood	Cohort studies	1940	OR: 3.02	1.34–6.83	Magnetic resonance	[20]
								Netherlands
	No other associated factors	Adults: middle age	Cohort studies	14,845	HR: 1.23	1.08–1.40 *p* < 0.0001	Ultrasound	[21]
								China

Gestational diabetes	Overweight or obesity during pregnancy	Adults: middle age	Cohort studies	1115	OR: 2.29	1.23–4.27 p 0.01	CT scan	[22]
								EE UU
			Cases and controls	40,936	RR: 2.46	1.51–4.00	Ultrasound	[23]
								United Kingdom
		Children: adolescence: young adult	Cases and controls	882	OR: 3.26	1.72–6.19	Liver biopsy	[24]
								Sweden

Thyroid disorders	Autoimmune thyroiditis	Adults	Cases and controls	114,966	OR: 1.53	1.35–1.73	Ultrasound	[25]
					*p* < 0.001			Germany
	Hypothyroidism		Cases and controls		OR: 1.17	1.10–1.24	Ultrasound	
					*p* < 0.001			
	FT3 > 5.34 pmol/L	Adults	Cohort studies	3144	OR: 2.00	1.70–2.36 *p* < 0.001	Ultrasound	[26]
								China

Psoriasis	Early-onset moderate to severe psoriasis	Adults	Cross-section	439	HR: 2.11	1.55–2.87	Ultrasound	[27]
								China
	Psoriasis managed with systemic therapy	Adults	Cases and controls	1,543,443	HR: 2.23	1.73–2.87	Ultrasound	[28]
	Psoriasis managed with systemic therapy	Adults			HR: 2.11	1.55–2.87		United Kingdom
	Severe psoriasis in adults with HIV infection	Adults	Cohort studies	5452	OR: 12	1.2–120 p 0.03	Ultrasound	[29]
								Spain

Polycystic ovary syndrome	Hyperandrogenism	Adults	Cases and controls	50,785 354	OR: 4.30	4.11–4.50 *p* < 0.001	Ultrasound	[30]
								EE UU
		Adults	Cases and controls	51	OR: 3.7	0.6–13.1 *p* < 0.05	Ultrasound	[31]
								United Kingdom

Increased uric acid levels	Obesity	Adults	Cross-section	4323	OR: 2.054	1.44–2.92 *p* < 0.0001	Ultrasound	[32]
								China
Men. ≥ 420 mol/L	Women							
Women. ≥ 360 mol/L	Postmenopausal women							
	No other associated factors	Adults	Cohort studies	24,102	HR: 1.66	1.28–2.15	Ultrasound	[33]
								China
	No other associated factors	Adults	Cross-section	60,455	F. HR: 2,35	F. 1.70–3.25	Ultrasound	[34]
					M. HR: 1.24	M. 0.97–1.60		China
	Obesity	Adults	Cohort studies	16,839	F. OR: 5.71	F. 2.25–14.45	Ultrasound	[35]
					M. OR: 1.99	M. 1.15–3.45		China
						*p* < 0.01		

Lifestyle	Consumption of carbonated drinks	Adults	Cross-section	7516	OR: 1.99	1.29–3.05 p 0.02	Ultrasound	[36]
								China
	Sedentary lifestyle	Adults	Cross-section	79,048	HR: 1.07	1.02–1.13	Ultrasound	[37]
								Korea
	Long working hours (> 41 h/week)	Adults	Cross-section		OR: 1.06	0.87–1.30 p 0.55	Ultrasound	[38]
								Korea
	Passive smoking + obesity and/or dyslipidemia	Childhood	Cohort studies	1315	RR: 1.04	1.02–1.07	Ultrasound	[39]
		Adults			RR: 1.14	1.05–1.25	Ultrasound	Finland
	Tooth brushing	Adults	Cross-section	4259	OR: 2.26	1.22–4.19 p 0.064	Ultrasound	[40]
								Korea
	Sleep quality	Adults	Cross-section	45,293	M. OR: 1.28	M. 1.13–1.44	Ultrasound	[41]
					F. OR: 1.71	F. 1.38–2.13		Korea
	≤ 5 h of sleep							
	Sleep quality				M. OR: 1.10	M. 1.02–1.19	Ultrasound	
					F. OR: 1.36	F. 1.17–1.59		
	Poor sleep quality							
	Smoking	Adults	Cohort studies	3860	OR: 1.98	1.057, 3.595	Ultrasound	[42]
								Japan

**Table 2:** The search strategy.

Question	MeSH term	Strategy
Population	“Adolescent”	(“Adolescent”) OR (“adult”) OR (“adult, young”) OR (“middle aged”) OR (“aged”) OR (“aged, 80”)
	“Adult”	
	“Adult, young”	
	“Middle aged”	
	“Aged”	
	“Aged, 80”	

Intervention	“Risk factor”	(“Risk factor”) OR (“risk factors”) OR (“genetic factors”) OR (“cardiovascular risk factor”) OR (“metabolic syndrome”) OR (“lifestyle factors”) OR (“physical inactivity”) OR (“sedentary lifestyle”) OR (“anthropometric”) OR (“environmental factors”) OR (“sociodemographic”) OR (“social determinants of health”)
	“Risk factors”	
	“Genetic factors”	
	“Cardiovascular risk factor”	
	“Metabolic syndrome”	
	“Lifestyle factors”	
	“Physical inactivity”	
	“Sedentary lifestyle”	
	“Anthropometric”	
	“Environmental factors”	
	“Sociodemographic”	
	“Social determinants of health”	

Outcome	“Non-alcoholic fatty liver disease”	(“Non-alcoholic fatty liver disease”) OR (“NAFLD”) OR (“metabolic dysfunction-associated fatty liver”) OR (“MAFLD”) OR (“nonalcoholic steatohepatitis”) OR (“NASH”) OR (“liver steatosis”) OR (“fatty liver”)

## Data Availability

The data that support the findings of this study are available from the corresponding author upon reasonable request.
